# Micronucleus count in nasal epithelial cells from patients with chronic rhinosinusitis and polyps^☆^

**DOI:** 10.1016/j.bjorl.2019.05.004

**Published:** 2019-06-18

**Authors:** Renata Loss Drummond, Cláudia Ramos Rhoden, José Faibes Lubianca Neto, Alan da Silveira Fleck, Rita Carolina Pozzer Krumenauer Padoin, Sérgio Luis Amantéa

**Affiliations:** Irmandade Santa Casa de Misericórdia de Porto Alegre, Hospital Santa Clara, Porto Alegre, RS, Brazil

**Keywords:** Micronucleus tests, Nasal polyps, Sinusitis, Inflammation, Teste micronúcleo, Pólipos nasais, Sinusite, Inflamação

## Abstract

**Introduction:**

Chronic rhinosinusitis with nasal polyps, a prevalent disease affecting around 2% of the world population, is characterized by symptomatic inflammation of the nasal mucosa and impairment of quality of life. Chronic rhinosinusitis with nasal polyps has a multifactorial etiology, involving a dysfunctional host response to environmental factors. Thus, inflammatory models may be useful to shed light on the pathophysiology of this disease. Micronucleus count has been used to screen DNA damage in various tissues.

**Objective:**

To investigate the association between frequency of micronucleus in exfoliated cells from the nasal cavity of patients with chronic rhinosinusitis with nasal polyps and disease severity.

**Methods:**

This cross-sectional study included 21 patients with chronic rhinosinusitis with nasal polyps and 19 controls without disease. None of the participants were smokers.

**Results:**

Mean micronucleus count was 3.690 per 1000 cells (±2.165) in individuals with vs. 1.237 per 1000 cells (±0.806) in controls; (Student's *t* test = 4.653, *p* < 0.001). Nasal surgery in the past 5 years and aspirin-exacerbated respiratory disease were not associated with nicronucleus count (*p* = 0.251).

**Conclusion:**

Micronucleus count seems to be linked to chronic rhinosinusitis with nasal polyps, providing a new perspective for the evaluation of this disorder.

## Resumo

**Introdução:**

A rinossinusite crônica com pólipos nasais, doença prevalente que afeta cerca de 2% da população mundial, é caracterizada por inflamação sintomática da mucosa nasal e comprometimento da qualidade de vida. A rinossinusite crônica com pólipos nasais tem etiologia multifatorial, envolvendo resposta disfuncional do hospedeiro a fatores ambientais. Assim, modelos inflamatórios podem ser úteis para esclarecer a fisiopatologia dessa doença. A contagem de micronúcleos tem sido usada para rastrear danos no DNA em vários tecidos.

**Objetivo:**

Investigar a associação entre a frequência de micronúcleos em células esfoliadas da cavidade nasal de pacientes com rinossinusite crônica com pólipos nasais e a gravidade da doença.

**Método:**

Estudo transversal que incluiu 21 pacientes com rinossinusite crônica com pólipos nasais e 19 controles sem doença. Nenhum dos participantes eram fumantes.

**Resultados:**

A contagem média de micronúcleos foi de 3,690 por 1.000 células (±2,165) nos indivíduos doentes e 1,237 por 1.000 células (±0,806) nos controles (Teste *t* de *Student* = 4,653; *p* < 0,001). A cirurgia nasal nos últimos 5 anos e a doença respiratória exacerbada por aspirina não foram associadas à contagem de micronúcleos (*p* = 0,251).

**Conclusão:**

A contagem de micronúcleos parece estar ligada à rinossinusite crônica com pólipos nasais, proporcionando uma nova perspectiva para a avaliação dessa doença.

## Introduction

Chronic Rhinosinusitis with Nasal Polyps (CRSwNP) is characterized by persistent (12 weeks or more) symptomatic inflammation of the nasal mucosa and paranasal sinuses, with development of nasal polyps.^1^ The worldwide prevalence of CRSwNP has been estimated to range from 2 to 5%.^2^, ^3^ In some patients, CRSwNP is associated with a major decline in quality of life, comparable to that resulting from rheumatoid arthritis and diabetes mellitus.^4^

A multifactorial etiology has been established for CRSwNP, possibly involving a dysfunctional host response to environmental factors as well as genetic and epigenetic contributions to increased individual susceptibility.^5^ Adequate clinical and/or surgical management of CRSwNP is known to improve quality of life significantly.^6^ However, for unknown reasons, in some patients CRSwNP is refractory to treatment, leading to persistence of symptoms and recurrence that is difficult to manage. Until the present moment, no laboratory resources are available to distinguish between different types of abnormalities or establish the prognosis of patients with CRSwNP.

In some respiratory disorders, such as asthma and acute viral bronchiolitis,^7^ Micronuclei (MN) have been identified as useful markers of cell damage. MN are capable of signaling events associated with cellular and molecular genotoxicity, providing support for a better understanding of etiology and pathogenesis, and consequently for the establishment of prevention strategies.^8^ Thus, the identification of MN in exfoliative cells of individuals with CRSwNP may be a useful tool to shed light on the mechanisms underlying CRSwNP.^9^

The objective of the present study was to investigate the association between frequency of MN in exfoliated cells from the nasal cavity of patients with CRSwNP and disease severity.

## Methods

A controlled cross-sectional study was carried out at the Otorhinolaryngology Clinic at Santa Casa de Porto Alegre. Using a consecutive sampling technique, we selected patients aged ≥18 years, with clinical diagnosis of CRSwNP, who had a medical appointment at the Otorhinolaryngology Service in July and August of 2015. Current smokers and patients with active infections were excluded.

A control group was chosen by convenience sampling at the hospital's surgical ward staff and relatives/individuals accompanying surgical patients were invited to participate. The same exclusion criteria used for patients were applied to the control group. In addition, those with active upper airway disease were also excluded. Individuals aged ≥18 years who agreed to participate in the study were included in the control group.

All participants provided written informed consent prior to the start of the study. The protocol was approved by the Research Ethics Committee at Santa Casa de Porto Alegre (8064/2015).

### Study protocol

Following medical interview and ENT examination, patients with evidence of CRSwNP were invited to join the study. Those who agreed to participate signed the informed consent form and answered a standardized health questionnaire, which was administered to all participants by one of the investigators (RLD), who also harvested exfoliative nasal mucosa cells for MN count. Exfoliate cells were collected with the patient in the seated position.

The procedure of specimen collection and analysis was based on the protocol described by Thomas et al.^10^ Briefly, specimens were collected from the middle meatus using a cytobrush and stored at 4 °C in Methacarn solution (methanol 3:1 acetic acid) for fixation (no longer than 14 days). In the laboratory, the microtubes were centrifuged for 5 min at 19 °C and 1000 RPM. After each centrifugation step, the specimens were rinsed three times and resuspended. After the last centrifugation step, 0.5 mL cell suspension aliquots were transferred to slides (two slides per individual) for staining.

The slides were sequentially stained: ethanol 50% for 1 min followed by ethanol 20% for 1 min and rinsing with distilled water for 2 min. The specimens were then immersed in 5 M HCl solution (40 mL HCI and 60 mL distilled water), rinsed with distilled water for 2 min and stained with Schiff's reagent for 80 min in a dark environment. After this period, the samples were rinsed with running tap water for 5 min and with distilled water for 1 min, followed by counterstaining with 0.2% fast green solution for 2 min. Finally, the samples were rinsed with distilled water for 2 min.

Specimens were analyzed using an optical microscope at 400× magnification. Only basal and differentiated cells were considered for analysis. The number of micronuclei was counted in 1000 cells per slide. The results were expressed as mean ± standard deviation. Normality of distribution was confirmed by the Shapiro–Wilk test. Student's *t* test was used for comparison of means, and the association between the variables was determined by the Chi-square test. A level of significance of 95% (*p* < 0.05) was adopted. The analyses were performed in SigmaPlot™ (version 13, 2012).

## Results

The sample included 40 participants, distributed into two groups according to the presence of polyps: 21 patients with polyps and 19 control patients without polyps. There were no differences between the groups regarding demographic aspects, except for age, which was higher in patients with CRSwNP (Table 1). Because Samter's triad (also known as Widal syndrome or aspirin-exacerbated respiratory disease, AERD) has been linked to a poor prognosis for sinus disease (1), we tested the association between MN count and AERD. MN was not increased in patients with AERD (*p* = 0.310). There was also no association between nasal surgery in the previous 5 years and MN count (*p* = 0.251).

**Table 1:** Characteristics of individuals with and without nasal polyps (controls).

	With polyps (*n* = 21)	Control (*n* = 19)	*p*
Age (years ± DP)	52.57 ± 9.79	41.05 ± 11.93	0.002^a^
Sex			
Male	10	9	0.763^b^
Female	11	10	
Rhinitis	14	13	0.826^b^
Asthma	8	6	0.921^b^
Alcohol abuse	1	0	1.000^c^
Previous sinus surgery <5 years	8	0	0,251^c^

a Student's t test.

b Chi-square test.

c Fisher's exact test.

**Figure 1:**
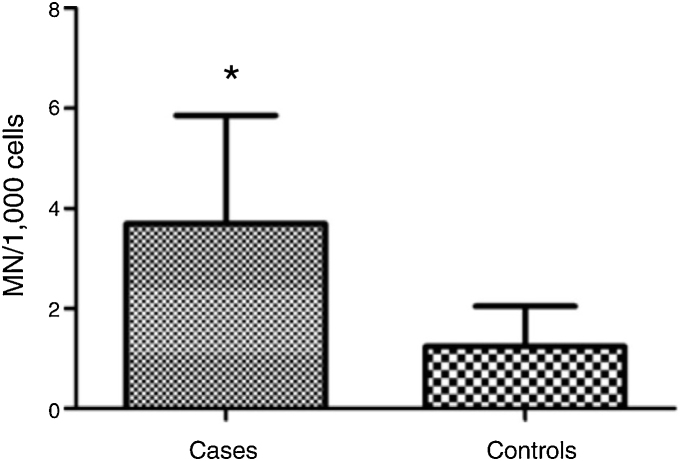
Number of micronuclei (MN) per 1000 cells in nasal exfoliative material of individuals with nasal polyps; (*p < 0.001).

**Figure 2:**
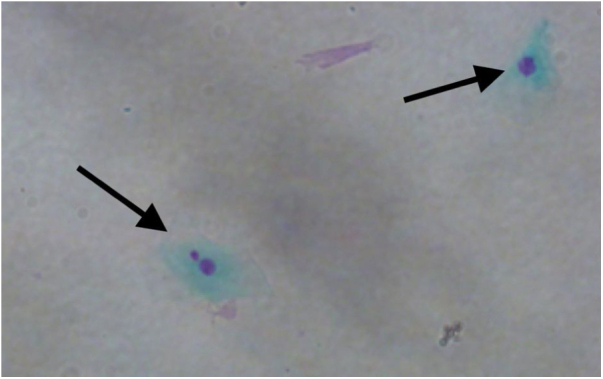
Micronuclei (left arrow) and normal nucleus (right arrow) in oral exfoliated epithelial cells (×400).

MN count was approximately three times higher in exfoliative cells of patients with polyps vs. controls: 3.690 ± 2.165 MN per 1000 cells vs. 1.237 ± 0.806 MN per 1000 cells respectively (*p* < 0.001) (Fig. 1). Fig. 2 shows a photomicrograph of MN in nasal exfoliated cells.

## Discussion

MN count in nasal mucosa cells obtained from carriers of CRSwNP and controls by an exfoliative technique was feasible, simple, and safe. The collection of exfoliative cells allows direct assessment of the target organ;^11^ for that reason, it has been used in studies focusing on a variety of diseases.^12^ MN have long been considered as carcinogenic biomarkers. In the past decades, MN have also been used as biomarkers of chromosomal damage, genomic instability, and risk of câncer.^13^ More recently, MN have been used to evaluate nasal mucosa damage, through the investigation of tissue genotoxicity in environmental monitoring studies.^14^

In the present study, the difference between participants with and without polyps in MN count – which was approximately three times higher in individuals with polyps – suggests cellular damage (genotoxicity) in carriers of CRSwNP. There have been no studies so far evaluating MN count in patients with nasal polyposis; nevertheless, other types of cell damage have been associated with nasal polyps.^15^, ^16^ Also, the present findings converge with data described in the literature for other diseases in which a higher MN count has been reported in the target cells of diseased patients as compared to disease-free controls.^11^ Mandard et al.^17^ found an MN count that was twice as high in the oral mucosa of digestive tract patients vs. controls. Chakrabarti and Dutta^18^ reported an increase in MN count that was proportional to the degree of malignancy detected in routine cervical smears. Herrström et al. observed increased MN count in blood samples of asthmatic youth.^19^ Recent studies also show that MN count is useful for biomonitoring of various disease stages, including genotoxicity screening, detection of malignancies, paraneoplastic disease, and effects of radiotherapy.^11^ However, some authors point to the need of establishing normal MN count thresholds in different tissues,^20^ which would facilitate standardization and comparison of results across studies worldwide.

We were unable to determine a correlation between the factors considered to have a negative impact on prognosis, such as AERD and surgery in the past 5 years, with increased MN count in exfoliative samples. We believe that this is a consequence of the small sample size, since previous authors have reported that MN count varies in different stages of disease, with a decrease in MN count in bladder cells after treatment of schistosomiasis^21^ as well as after the use of DNA-protective agents such as beta-carotene and vitamin A.^22^

Our study has some limitations, such as small sample size. The inability to determine differences between the groups, especially regarding prognostic factors, may have resulted from low sample power, leading to type II error. Also, the age difference between patients with polyps and controls may have introduced some bias. Nevertheless, both groups included patients with the same biological status, most of which were adults or older adults.^23^ Recent studies^24^, ^25^ have suggested that MN count is increased in older adults. Conversely, investigators such as Calderon-Garciduenas et al.^26^ have not observed differences in MN count associated with age. Thus, larger studies may contribute to clarify the role of age in MN prevalence.

## Conclusion

In conclusion, MN count in exfoliative nasal mucosa cells from patients with CRSwNP was feasible, simple, and safe. This technique does not require sophisticated laboratory resources, and can be easily performed in both experimental and clinical settings. We believe that the increased MN count found in our patients with CRSwNP provides a new perspective for the evaluation of this disorder, whose pathophysiology has not yet been fully understood.

## Funding

Approved at Santa Casa de Porto Alegre Ethics Committee (CEP-ISCMPA) — n° 8064/2015.

## Conflicts of interest

The authors declare no conflicts of interest.
